# Alignment of Qx100/Qx200 Droplet Digital (Bio-Rad) and QuantStudio 3D (Thermofisher) Digital PCR for Quantification of BCR-ABL1 in Ph+ Chronic Myeloid Leukemia

**DOI:** 10.3390/diseases9020035

**Published:** 2021-05-05

**Authors:** Carmen Fava, Simona Bernardi, Enrico Marco Gottardi, Roberta Lorenzatti, Laura Galeotti, Francesco Ceccherini, Francesco Cordoni, Filomena Daraio, Emilia Giugliano, Aleksandar Jovanovski, Jessica Petiti, Marta Varotto, Davide Barberio, Giovanna Rege-Cambrin, Paola Berchialla, Veronica Sciannameo, Michele Malagola, Giuseppe Saglio, Domenico Russo

**Affiliations:** 1 Department of Clinical and Biological Sciences, University of Turin, AOU San Luigi Gonzaga, Orbassano, 10043 Turin, Italy; aleksandar.jovanovski@unito.it (A.J.); jessica.petiti@unito.it (J.P.); paola.berchialla@unito.it (P.B.); giuseppe.saglio@unito.it (G.S.); 2 Unit of Blood Diseases and Stem Cell Transplantation, DPT of Clinical and Experimental Sciences, University of Brescia, ASST SpedaliCivili di Brescia, 25123 Brescia, Italy; simona.bernardi86@gmail.com (S.B.); michelemalagola@yahoo.it (M.M.); domenico.russo@unibs.it (D.R.); 3 Division of Internal Medicine and Hematology, San Luigi Gonzaga Hospital, Orbassano, 10043 Turin, Italy; enricogottardi@libero.it (E.M.G.); roberta.lorenzatti@gmail.com (R.L.); filodaraio@tiscali.it (F.D.); e.giugliano@sanluigi.piemonte.it (E.G.); giovanna.rege@libero.it (G.R.-C.); 4 PhymtechSrl, Via F.lli Rosselli 8, Madonna dell’Acqua, San Giuliano Terme, 56017 Pisa, Italy; laura.galeotti@gmail.com (L.G.); francesco.ceccherini@gmail.com (F.C.); f.cordoni@phymtech.com (F.C.); 5 Bioclarmas.r.l. Research and Molecular Diagnostics, 10126 Torino, Italy; marta.varotto@bioclarma.com (M.V.); davide.barberio@bioclarma.com (D.B.); 6 Unit of Biostatistics, Department of Cardiac, Thoracic and Vascular Sciences, Epidemiology and Public Health, University of Padova, 35128 Padova, Italy; veronica.sciannameo@unito.it

**Keywords:** digital polymerase chain reaction, digital PCR, dPCR, chronic myeloid leukemia, CML, minimal residual disease

## Abstract

In recent years, the digital polymerase chain reaction has received increasing interest as it has emerged as a tool to provide more sensitive and accurate detection of minimal residual disease. In order to start the process of data alignment, we assessed the consistency of the BCR-ABL1 quantification results of the analysis of 16 RNA samples at different levels of disease. The results were obtained by two different laboratories that relied on The Qx100/Qx200 Droplet Digital PCR System (Bio-Rad) and Quant Studio 3D dPCR System (Thermofisher) platforms. We assessed the compatibility between the estimated values by linear regression, Bland–Altman bias-plot, and Mann–Whitney nonparametric test. The results confirmed the compatibility of the measures, allowing us tocompute an ‘alignment factor’ (AF), equal to 1.41, which was further validated by a different series of experiments. We conclude that the performed measurements by the two laboratories are comparable, and also equalized through the introduction of an alignment factor.

## 1. Introduction

Recent long-term survival estimates of chronic myeloid leukemia (CML) patients treated with tyrosine kinase inhibitors (TKIs) show that life expectancy for these patients is increasing to almost that of the general population [1,2,3]. This means that molecular monitoring in these patients must be brought to a level that will give early risk stratification, better prognoses, and a more accurate decision as to whether to suspend the TKI treatment. The current strategy of CML treatment with TKIs is aimed at achieving at least a major molecular response (MMR) to prevent progression to the blastic phase (BP) and possibly reaching a deep molecular response (DMR), raising the opportunity for treatment discontinuation [4,5,6]. In fact, recent trials have demonstrated that a consistent percentage of CML patients who have achieved stable DMR for a sufficient period can safely stop their therapy without relapsing [7], and treatment-free remission (TFR) has consequently become a goal for treatment based on TKIs. 

Real-time PCR (RT-qPCR) is currently used as a standard test for laboratory diagnosis and assessing molecular response (MR) in CML patients. However, despite the international efforts to standardize the method, RT-qPCR still has some intrinsic limitations to itsaccuracy and sensitivity. Although the depth of MR is not the only element utilized to predict a successful TFR, it is recognized that a more sensitive and accurate method for the detection of minimal residual disease (MRD) would represent an advantage for patients aiming to achieve TFR.

In recent years, digital PCR (dPCR) has emerged as a possible alternative for RT-qPCR. It appears able to provide more sensitive and reproducible detection of very low levels of disease and such a capability has generated an increased interest inits potential utilization in clinical practice [8,9]. From a technical point of view, dPCR is a third-generation PCR that provides an end-point measurement of the target, partitioned in reaction chambers within specially designed chips or throughout an oil-water emulsion resulting in thousands of individual PCR reactions. A Poisson correction is applied to estimate an absolute target sequence quantity, without the need for a standard curve [10,11,12,13,14].

Despite itsapparent advantages, dPCR is not yet used routinely. Also, several of dPCR’s key features, as well as related biomedical applications and perspectives, are still under investigation. Preliminary data have provided indications that dPCR exhibits higher sensitivity in monitoring MRD and higher accuracy in identifying patients with higher probabilities of relapse after discontinuation of TKIs [15,16,17,18]. Although these studies have shed light on dPCR’s potential, standardization of the method will surely provide a broader and more general application of dPCR. 

At present, there are various dPCR platforms available with different characteristics and technical specifications, e.g., Qx100/200 (Biorad), which has adroplet-based workflow, and QuantStudio 3D Digital PCR System (Thermofisher),which is based on a chip workflow. The QX200 Droplet Generator is used to partition the reaction mix and target about 20,000 nanoliter-sized droplets. After the amplification on a thermal cycler, droplets are analyzed individually with a two-color optical detection system in a serial manner. The QuantStudio™ 3D Digital PCR Instrument makes a physical type of partition on a chip obtaining about 20,000 reaction wells. The instrument performs multiple image captures of the chip and, after the run, it determines the location and intensity of the fluorescent signals in each image.Up to 96 samples can be processed per run using the BioRad platform, while 24 samples can be processed simultaneously using the ThermoFisher platform. The PCR-positive and negative droplets are counted to provide absolute quantification of the target.Both QX200 Droplet Digital PCR System and QuantStudio 3D Digital PCR System can work with both Probe-Based and EvaGreensystems.Performances have to be defined assay by assay for both platforms. For QX200 Droplet Generator, the cost is slightly higher than Real-Time PCR, and the whole process from cDNA to the final resultstakesabout 5 h. 

In this study, we aimed to compare the results of the quantification of a p210 BCR-ABL1 transcript obtained from two different laboratories thatused two different dPCR platforms in order to: (i) to assess the consistency of the results; and (ii) to verify and validate the possible existence of an ‘alignment factor’ (AF) between the two platforms.

## 2. Materials and Methods

### 2.1. Sample Characteristics, RNA Extraction and cDNA Synthesis

With the approval of the institutional ethical committee, RNA samples of 16 Ph+ CML patients (4 samples of peripheral blood for each level of BCR-ABL1/ABL1 percentage measured by RT-qPCR: 10–1%, 1–0.1%, 0.1–0.01%, <0.01%) were extracted with a Maxwell^®^ 16 instrument (Promega, Madison, WI, USA) using SimplyRNA Blood Kit LEV (Cat.# AS1310), according to the manufacturer’s recommendations. We decided to test samples with a transcript level of <10% (chronic phase CML) because of the advantage in the accuracy of dPCR in lower levels of the disease [8,9]. The samples were quantified by using the NanoDrop™ One/OneC Microvolume UV-Vis Spectrophotometer (ThermoFisher Scientific, Waltham, MA, USA) and then reverse-transcribed in 4 reactions (RT) performed on different weeks. Synthesis of complementary DNA (cDNA) was performed by adding 3µg of RNA to a 50 µL mix composed by 200U of MuLV Reverse Transcriptase (AB Cat No. N8080018) and Hexanucleotide Primers (Sigma, Merck, Munich, Germany, Cat No H0268) at aconcentration of 25 M according to this thermal profile: 20 °C for 10′, 42 °C for 120′, 99 °C for 3′ and 4 °C∞. All the RNAs were extracted and reverse transcribed centrally in one laboratory and then cDNAs were shared with the second laboratory. Finally, cDNA products were tested in 4 different dPCR runs (hereafter, Exp1, Exp2, Exp3, and Exp4) from the two involved laboratories (hereafter, Lab 1 and Lab 2). 

The first experiment (Exp1) was realized by testing a larger number of BCR-ABL1 replicates for 8 out of the 16 samples for each level of disease (10 replicates for the Qx100/Qx200 Droplet Digital dPCR approach and 6 replicates for the QuantStudio 3D Digital PCR System). For each sample, all possible combinations of replicates were considered. ABL1 was tested for each replicate as a control gene.

The additional three experiments, named Exp2, Exp3, and Exp4 respectively, were conducted on the whole set of 16 samples by the two laboratories. For each sample and experiment, Lab 1 tested BCR-ABL1 in triplicates and ABL1 in duplicates, whereas Lab 2 tested both BCR-ABL1 and ABL1 in duplicates. 

### 2.2. Digital PCR Platforms and Analysis 

#### 2.2.1. Lab1: Qx100/Qx200 Droplet Digital PCR System

Experiments were performed in a singleplex utilizing the QX100™ Droplet Digital PCR System platform (Bio-Rad, Hercules, CA, USA) operating with the DigiDrop p210 Master Mix Kit (BioclarmaS.r.l, Turin, Italy), according to the manufacturer’s instructions. Two different positive controls, 1% and 0.01%, and a negative control (DNA- and RNA-free water) were also included in each analysis. The target gene BCR-ABL1 p210 was analyzed in triplicate using 200 ng/replicate of RNA Equivalent [RNAEq represents the amountof the RT reaction product (cDNA) estimated from the amount of initial RNA], while the reference gene ABL1 was tested in duplicate using 100 ng/replicate of RNAEq for both samples and controls. The plate underwent thermocycling following specific amplification conditions, indicated by the manufacturer: 95 °C for 10′, 45 cycles at 94 °C for 30″ and 60 °C for 1′, followed by a final extension step at 98 °C for 10′. Threshold values were set at 4000 for BCR-ABL1 p210 and 8500 for ABL1, according to the manufacturer’s instructions. The sample that revealed <8000 analyzed droplets (minimum droplets number to validate the results) or copy numbers for reaction >60,000 (saturation of the system) were excluded from the subsequent data analysis, according to the manufacturer’s instruction. The limit of detection (LOD), the limit of blank (LOB), and the threshold values are indicated by the producer. The BCR-ABL1 and ABL1 copy numbers were then used to calculate the percentage of BCR-ABL1/ABL1, according to the European Against Cancer Program and the latest EUTOS recommendations [4].

#### 2.2.2. Lab2: QuantStudio 3D Digital PCR System 

Experiments were performed in singleplex by the QuantStudio 3D Digital PCR System platform (Thermofisher Scientific, MA, USA) using the QuantStudio 3D Digital PCR Master Mix V2 (Thermofisher Scientific, MA, USA), according to the manufacturer’s instructions. A negative control was also included in each analysis. The target gene BCR-ABL1 p210 was analyzed in duplicate using 50 ng/replicate of RNAEq, while the reference gene ABL1 was tested in duplicate using 25 ng/replicate of RNAEq for both samples and controls. An FAM-labeled assay targeting BCR-ABL1 and a VIC-labeled assay targeting ABL1 were custom designed. Theprimer and probe sequences were:

**BCR-ABL1 assay**

Forward primer: 5′ TCCGCTGACCATCAAYAAGGA 3′

Reverse primer: 5′ CACTCAGACCCTGAGGCTCAA 3′

Probe: 5′ TTCAGCGGCCAGTAGCAT 3′

**ABL1 assay**

Forward primer: 5′ ACTCTAAGCATAACTAAAGG 3′

Reverse primer: 5′ GATGTAGTTGCTTGGGACCCA 3′

Probe: 5′ AAGCCCAAACCAAAAAT 3′

We prepared 16 µL of reaction mix containing 8 µL of 2X QuantStudio 3D Digital PCR Master Mix (Life Technologies, Carlsbad, CA, USA), 0.8 µL of 20X TaqMan-MGB-FAM-probe assay, 1.1 µL of diluted cDNA (50 ng/µL), and 6.1 µL of nuclease-free water (Qiagen). For the quantification of positive controls, negative controls, and standard dilutions, 15 µL of the reaction mix were loaded onto a QuantStudio 3D Digital PCR 20 K Chip using the automatic chip loader according to the manufacturer’s instructions. The loading allows the subdivision of the reaction into20,000 micro-reactions, corresponding to the 20,000 micro-wells onto the surface of the chip. Every reaction has a final volume of 865 pL.

Loaded chips underwent thermo-cycling following specific amplification conditions: 95 °C for 8′, 45 cycles at 95 °C for 15″ and 60 °C for 1′, followed by a final extension step at 60 °C for 2′. The samples that revealed a low number of correctly loaded wells (<16,000) or copy numbers for reaction >15,000 (saturation of the system) were excluded from the subsequent data analysis [16]. The BCR-ABL1 and ABL1 copy numbers were then used to calculate the percentage of BCR-ABL1/ABL1, according to the European Against Cancer Program and the latest EUTOS recommendations [4]. 

The LOD was calculated by quantifying standard dilutions of plasmid containing BCR-ABL1 transcript sequence (QIAGEN) at a known concentration (10^6^ copies, 10^5^ copies, 10^4^ copies, 10^3^ copies, 10^2^ copies, 10 copies) at optimal thermocycling conditions, and the results were correctly quantified.

Ten replicates of negative controls obtained by quantifying DNA- and RNA-free water were used to determine the LOB of the test, calculated by multiplying by three the standard deviation of the measures (Standard Deviation of Blanks Response) [19]. LOB analysis was 0.066 BCR-ABL1 copies/uL, corresponding to 0.99 BCR-ABL1 copies/chip. This value was used to evaluate the maximum background noise and confirmed the specificity of this assay.

All the signals captured from negative controls were under 4000 Relative Fluorescence Unit (RFU) for BCR-ABL1 and 2500 for ABL1, so these values were the thresholds between positive and negative emissions. 

### 2.3. Statistical Analysis

Statistical analyses were performed following a multistep approach. First, linear regression was used to assess the compatibility between the values obtained in the two laboratories. Second, to confirm the compatibility between the results from the two laboratories, we applied the Branford method [19] and compared the measures via the Bland–Altman bias-plot limit of agreement [20,21,22,23]. Third, following the Bland–Altman method approach, we computed the AF value as the antilog of the average of the differences [20,21,23]. Finally, the obtained AF value was verified in terms of ‘fold-analysis’ [23].

## 3. Results

To assess the consistency of the results between Lab1 and Lab2, we analyzed data from a subset of the original 16 samples. In particular, 8 samples representing 2 samples of each disease level were selected (Exp1). These samples offered the highest number of replicates for both platforms, namely 10 replicates for the Qx100/Qx200 Droplet Digital dPCR (Bio-Rad) approach and 6 replicates for the QuantStudio 3D Digital PCR System (Thermo Fisher Scientific). Data were expressed as the ratio of BCR-ABL1/ABL1 in the percentage of every single replicate. The concentration of extracted RNA, raw result of RT-qPCR, and ddPCR are provided in Supplementary Table S1.

**Figure 1:**
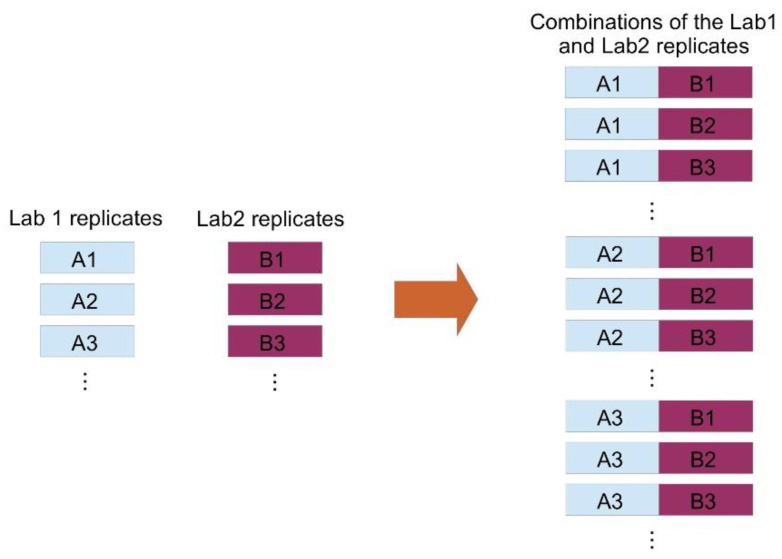
Method of combination of replicates from the twolaboratories. The set of measures iscreatedby combiningeach measure from Lab 1 (A) with each measure from Lab 2 (B).

All validated results were included in the analysis. This allowed us to avoid the additional uncertainties caused by the different mathematics used by the two laboratories to calculate the final results from the replicates’ values. For each sample, we considered all possible combinations of replicates from the two laboratories and we obtained a global set with 429 entries (see Figure 1). In the following, we will indicate with an ‘A’ each measure of BCR-ABL1/ABL1 % obtained from Lab1, and with a ‘B’ each measure of BCR-ABL1/ABL1 % obtained from Lab2. Therefore, each of the 429 combinations is formed by a measure A and a measure B.

With the help of linear regression, we checked the compatibility between the values obtained in the two laboratories and, as the value of the coefficient of determination (R^2^) we obtained 0.9869, which we consider very satisfactory. To confirm the compatibility between the two laboratories, we followed the Branford method and compared the measures through a technique based on the Bland–Altman bias-plot approach [20,21,22]. With respect to the original Bland–Altman approach, we used a slightly different criterion, i.e., in the bias plot we included all combinations of the replicates for each sample instead of the average values only. This leads to a stricter criterion. For the sake of completeness, it is worth noting that the original Bland–Altman method also includes a contribution due to the replicates but rather than being direct as in our case it is carried in through the “within subject standard deviation” [22]. The calculus of the AF is not affected by the use of all combinations of the replicates, but nevertheless our choice is significantly beneficial for the subsequent analyses as it allows to apply and verify stricter constraints on the variability between the measurements obtained in the two laboratories. More specifically, we request that the so-called “95% limit of agreement” [22] is satisfied by the majority of the measures and not only by the corresponding average values. 

**Figure 2:**
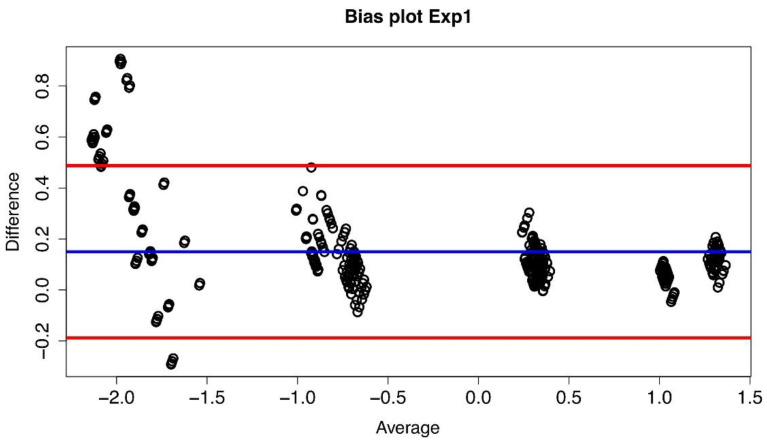
Bias plot of Lab1/Lab2 for Exp1. The difference between the two corresponding datasetsare plotted versus their average value. The blue line represents the mean value of thedifferences (d) (where each difference is defined as log_10_(B) − log_10_(A)), and the red lines define the 95% limits of agreement.

The bias plot we have obtained is shown in Figure 2, and it has been constructed considering the log10 of the measures and plotting for each of the 429 combinations the difference between the values obtained at Lab2 and Lab1, i.e., log_10_(B) − log_10_(A), versus the average value of that particular combination, i.e., (log_10_(B) + log_10_(A))/2. 

**Figure 3:**
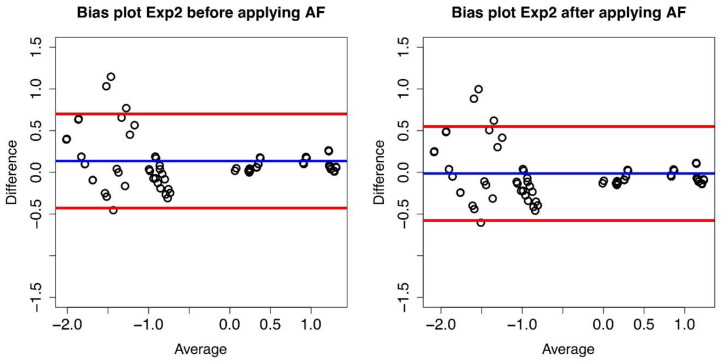
Bias plot of Lab1/Lab2 for Exp2. The difference between the two corresponding datasetsare plotted versus their average value.The blue line represents the mean value of thedifferences (d) (where each difference is defined as log_10_(B) − log_10_(A)), and the red lines define the 95% limits of agreement before conversion (**left** panel) and after conversion (**right** panel).

If the plot of the differences exhibits a non-zero mean value bias, it follows that between the values measured in the two laboratories there is a systematic difference (multiplicative in our case). Indeed, such a systematic difference between the two laboratories has been observed (see Figure 3), and it led to a confirmation of the compatibility between the measures obtained at Lab1 and Lab2 and to the possibility of computing an AF value thaatwas then found to be equal to 1.41 with a confidence of agreement range equal to [1.36–1.47].

We report in Table 1 the mean of the differences before and after the conversion and the standard deviation of the differences. Taking Lab1 as the reference laboratory, Lab2 results can be converted by multiplying by 1/AF and as expected, the mean of the differences after the conversion is zero.

The obtained AF value was then validated on a different series of experiments, namely, Exp2, Exp3, and Exp4. Again, we considered all the replicate combinations and we obtained 84, 86 and 88 combinations, respectively, and we used the AF to convert Lab2 dPCR% to Lab1-like dPCR%. The obtained results proved to be very satisfactory.

In order to demonstrate the benefit of the conversion, we used for each experiment the ‘fold difference’ approach. As in Muller et al. [23], we defined the ‘fold difference’ (FD) as the ratio of B/A, where A is the reference measure and B is the test measure, in our case Lab1 and Lab2, respectively. We computed the percentage of measures thatbefore and after conversion are included in a 2-fold range (with FD between 0.5 and 2), in a 3-fold range (0.33–3), and 5-fold range (0.2–5) (see Table 2). 

In general, one can state that there is an acceptable concordance between measures obtained in two different laboratories if at least two of the following three conditionsaremet [23]:

(a) more than 50% of the values lie within a 2-fold range;
(b) more than 75% of the values lie within a 3-fold range;
(c) more than 90% of the values lie within a 5-fold range.

The values reported in Table 2 show that our data meet all three conditions before and after the conversion with a general improvement after the application of the AF. 

**Figure 4:**
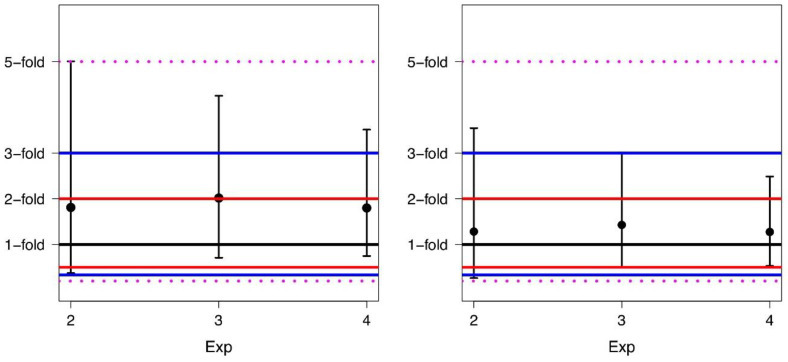
Means and corresponding 95% limits of agreement of the fold differences for Exp2, Exp3, and Exp4 before conversion (**left** panel) and after conversion (**right** panel).

For each experiment, we also computed the FD mean, median values, and the 95% limits of agreement before and after alignment (Table 3). A corresponding graphical description, including the fold range limits, is shown in Figure 4.

It is worth pointing out that an average difference of 1.0-fold indicates that there is no difference in the average values of BCR-ABL1/ABL1% obtained in the two laboratories. Comparing data, one can see that the conversion leads to a narrowing of the limits of agreement, i.e., the application of AF narrows the width of the data distribution. In particular, after conversion, the values of the 95% limits of agreement lie for all experiments in a 3.6-fold range rather than in a 5.0-fold range as before the conversion.

## 4. Discussion

In this study, we compared two dPCR platforms in order to evaluate the consistency of their data. In addition, we accounted for the possibility of calculating anAF to minimize the variability between the results obtained from the two platforms. So far, RT-qPCR is considered a gold standard for monitoring molecular response and diagnosis of CML patients. Its process of international standardization has been active for more than 20 years, going through all the stages of results validation, conversion factor implementation, and expressing the results on an international scale, as well as creating reference material for the proper quantification of BCR-ABL1 transcripts [4,20,23,24,25,26,27]. Despitemany efforts to standardize the method, RT-qPCR still carries considerable uncertainty, especially in detecting very low levels of the disease [28]. In particular, RT-qPCR presents some intrinsic defects, i.e., requirement of a standard curve for the quantification and loss of accuracy in case of a small number of leukemia cells.

The introduction of therapy with TKIs has drastically increased survival rates, determining the need to perform a more advanced molecular analysis to have a better definition of the deeper response levels of the disease [4]. On the other hand, by defining DMR and introducing additional MR4 and MR4.5 levels of the disease, the shortcomings of RT-qPCR are highlighted when it comes to long-term follow-up of CML patients. The current policy of CML treatment with TKIs is aimed atachieving an MMR to prevent progression to advanced phases [29,30,31,32] and a stable DMR to provide a chance for TFR [33,34]. Therefore, all scientific approaches are focused on finding the most appropriate method and meeting the fully or partially set criteria, which is essential for clinical management.

The dPCR is certainly not a completely new method, but the interest in its application in the hematology field has beenespeciallypopularin recent years. A newly released review by Cilloni et al. has summarized the characteristics and advantages of dPCR over RT-qPCR in the possible future application for monitoring not only the CML patients but also patients with other hematological disorders [35]. From a methodological point of view, dPCR does not require the utilization of reference material or standard curves, resulting in higher reproducibility. The Qx100/Qx200 Droplet Digital PCR System (Bio-Rad) and QuantStudio 3D Digital PCR System (Thermo Fisher Scientific) are the most widespread platforms commonly used in Italy. These instruments present similar features, such as a maximum partition number of 20,000 and the possibility of expressing results by the same units of measurement. However, they are based on two different receptacles: Qx100/Qx200 (Bio-Rad) is based on sample subdivision in micro-droplets, while QuantStudio 3D (Thermo Fisher Scientific) is a chip-based platform, which presents a physical division of the sample in micro-wells [8,28,36]. Although guidelines for the use of the dPCR have been published, no standardization process has yet been implemented.

Our study results revealed a satisfactory level of accuracy and reproducibility, confirming the value of the dPCR technique. Although we observed higher variability in the quantification of the lower disease levels, we concluded that the two used dPCR platforms have shown consistent results. Despite the two platforms using different RNA amount for the analysis, the workflow is optimized in order to have the best results for both platforms.In fact, the differences in the variability are independent of the amount of equivalent RNA used for the analysis.

The high reproducibility of dPCR, however, does not make it exempt from the need for a standardization process. Indeed, our data highlighted that two different dPCR platforms also need the introduction of an AF to make the results completely comparable. The main strengthof our study lies in demonstrating the possibility of using two different highly effective dPCR platforms with comparable results, thanks to the introduction of an AF. The demonstration of how useful an AF can be, even for very reproducible systems, such as dPCR, underlines the importance of undertaking a standardization path similar to that carried out in RT-qPCR.

Our study had some limitations. First, even though these are promising and satisfactory results, they are preliminary and cannot give a definitive picture of what our original aim was at the beginning ofthe study. To answer that question in full requires long-term comparative studies and the collaboration of several laboratories using the described dPCR platforms. More comprehensive studies for confirmation of the dPCR’s superiority over RT-qPCR are also needed, and that could be achieved by directly comparing the results from these two methods [10,37,38,39]. Second, we used only a total number of 429 replicates for the main study analysis, but still following the Bland–Altman method approach from a mathematical point of view is possible to apply it satisfactorily. Especially, considering that the total variance is mostly given by the within-subject variance and, in this way, the leading terms of the variance are properly represented.

The results presented here are part of an ongoing study for the evaluation of the variability of the measurements of different levels of CML disease within the same laboratory and among 5 different laboratories. We strongly believe that such an investigation in the future will increase the interest in applying dPCR for molecular monitoring of hematology diseases, and that this will provide better risk stratification and a more accurate prognosis. According to the results of our study so far, the dPCR method can be examined as a reasonable alternative to theRT-qPCR method, and its standardization process could be taken as feasible and achievable.

## Figures and Tables

**Table 1:** Standard deviation (σ) and mean of the differences (d) before and after conversion for Exp1, Exp2, Exp3, and Exp4.

	Standard Deviation (σ)	d before AF	d after AF
Exp 1	0.17	0.150	0.000
Exp 2	0.29	0.135	–0.014
Exp 3	0.20	0.240	0.090
Exp 4	0.17	0.209	0.060

**Table 2:** Percentages of measurements, before and after conversion, that lie within a 2-, 3-, and 5-fold range for Exp2, Exp3, and Exp4 presented under (A), (B), and (C) respectively.

(A)			
Exp 2	2-Fold	3-Fold	5-Fold
Before conversion	79.1	87.2	96.5
After conversion	74.4	87.2	97.7
(B)			
Exp 3	2-Fold	3-Fold	5-Fold
Before conversion	78.6	94.0	95.2
After conversion	91.7	95.2	96.4
(C)			
Exp 4	2-Fold	3-Fold	5-Fold
Before conversion	80.7	95.4	96.6
After conversion	95.4	95.4	97.7

**Table 3:** Fold difference (FD) before (A) and after conversion (B). We report the 95% limits ofagreement, the mean of the ‘fold differences’ (FD = B/A), and the median value for each experiment.

(A)			
	Mean	95% Limits of Agreement	Median
Exp 2	1.81	0.37–5.01	1.16
Exp 3	2.01	0.71–4.25	1.69
Exp 4	1.80	0.74–3.51	1.40
(B)			
	Mean	95% Limits of Agreement	Median
Exp 2	1.28	0.26–3.54	0.82
Exp 3	1.43	0.50–3.01	1.20
Exp 4	1.27	0.53–2.49	0.99

## Data Availability

All relevant data are within the paper and its Supporting Information files (such as Supplementary Material).

## Supplementary Materials

The following are available online at https://www.mdpi.com/article/10.3390/diseases9020035/s1. The concentration of extracted RNA, raw result of RT-qPCR, and ddPCR.
